# Patient preference for involvement, experienced involvement, decisional conflict, and satisfaction with physician: a structural equation model test

**DOI:** 10.1186/1472-6963-13-231

**Published:** 2013-06-25

**Authors:** Lars P Hölzel, Levente Kriston, Martin Härter

**Affiliations:** 1 Division of Psychiatry and Psychotherapy, Clinical Epidemiology and Health Services Research, University Medical Center Freiburg, Freiburg, Germany; 2 Department of Medical Psychology, University Medical Center Hamburg-Eppendorf, Hamburg, Germany

**Keywords:** Patient Preference, Informed Choice, Decision-making, Patient-caregiver Communication, Patient Satisfaction

## Abstract

**Background:**

A comprehensive model of the relationships among different shared decision-making related constructs and their effects on patient-relevant outcomes is largely missing. Objective of our study was the development of a model linking decision-making in medical encounters to an intermediate and a long-term endpoint. The following hypotheses were tested: physicians are more likely to involve patients who have a preference for participation and are willing to take responsibility in the medical decision-making process, increased patient involvement decreases decisional conflict, and lower decisional conflict favourably influences patient satisfaction with the physician.

**Methods:**

This model was tested in a German primary care sample (N = 1,913). Psychometrically tested instruments were administered to assess the following: patients’ preference for being involved in medical decision-making, patients’ experienced involvement in medical decision-making, decisional conflict, and satisfaction with the primary care provider. Structural equation modelling was used to explore multiple associations. The model was tested and adjusted in a development sub-sample and cross-validated in a confirmatory sample. Demographic and clinical characteristics were accounted for as possible confounders.

**Results:**

Local and global indexes suggested an acceptable fit between the theoretical model and the data. Increased *patient involvement* was strongly associated with decreased *decisional conflict* (standardised regression coefficient Β = −.73). Both high experienced *involvement* (Β = .34) and low *decisional conflict* (B = -.28) predicted higher *satisfaction with the physician*. Patients’ *preference for involvement* was negatively associated with the *experienced involvement* (B = −.24).

**Conclusion:**

Altogether, our model could be largely corroborated by the collected empirical data except the unexpected negative association between preference for involvement and experienced involvement. Future research on the associations among different SDM-related constructs should incorporate longitudinal studies in order to strengthen the hypothesis of causal associations.

## Background

A crucial element of medical communication is the process of medical decision-making which has received serious attention in recent years [1]. The concept of shared decision-making (SDM) plays an essential role in this context. SDM describes an interaction process in which both patient and physician participate equally and actively in finding an agreement based on shared information. The objective is to reach a decision for which both patient and physician take responsibility [2].

Patient involvement plays a central role in primary care because primary care is often the source of first contact, and general practitioners have to collaborate with patients to identify their healthcare needs and choose corresponding services [3]. Evidence of the association between enhanced involvement and higher satisfaction is limited and inconclusive [4,5]. In addition, research findings show that patients wish to be more involved in medical decision-making [6].

Unique SDM-related constructs have been examined in numerous studies. In this context, constructs like preference for involvement in medical decisions, experienced involvement, decisional conflict, and patient satisfaction have been investigated [6-9] (see Table 1). However, a comprehensive model of the relationship between SDM and related constructs, empirical studies focusing on the associations between these constructs, and a better understanding of the key elements associated with SDM are missing thus far [5].

**Table 1:** Shared decision-making-related constructs

**Construct**	**Definition**
Preference for involvement in medical decision mak	Preference of a patient for active participation in decisions concerning a choice between medical treatment options. Low preference indicates no wish for involvement, while high preference refers to a wish for an active role in the decision-making process.
Experienced involvement	Degree to which a patient feels involved in the process of medical decision-making. It can also be defined as the patient’s impression as to the extent that a decision is “shared” between the patient and the physician. Low involvement signals a rather authoritative process controlled by the physician, while high involvement indicates a shared decision-making or even an autonomous decision by the patient.
Decisional conflict	Perceived conflict between medical treatment options. It provides information on the subjectively experienced quality of a decision that has been reached. Low conflict corresponds to a rather satisfactory decision, while high conflict indicates that the selected treatment option is not necessarily believed to be the best option and substantial doubts remain.
Patient satisfaction	Patient’s global satisfaction with the medical care provided by his or her physician.

**Figure 1:**
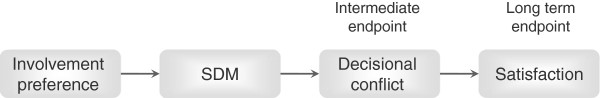
Conceptual model of central shared decision-making related constructs.

The present investigation was based on the assumptions of a conceptual model (Figure 1). In accordance with the measurement framework of Scholl et al. [10], we considered the levels of the decision antecedents, decision process, and decision outcome in our model. On the level of decision antecedents, we considered patient *preference for involvement in decision-making*, as it may play a crucial role in the communication process [10]. As a process indicator, we surveyed the *experienced involvement* of the patients in the clinical decision-making process. Following the recommendations of de Haes and Bensing, we differentiated between intermediate and long-term endpoints [11]. *Decisional conflict* has been recommended as an appropriate outcome of SDM [10] and is often used in outcome studies [12]. Decisional conflict is considered an optimal intermediate outcome, as the construct is closely related to the decision-making process. *Satisfaction with physician* is also a very common outcome of SDM in the literature [5,13]. As it is a rather general construct, satisfaction with physician was used as a long-term outcome.

The following hypotheses were generated:

•A high preference for being involved in medical decisions is linked to an increased involvement in decision-making.

•Stronger involvement diminishes the decisional conflict.

•Lower decisional conflict favourably influences satisfaction with the physician.

The primary question of this study was whether the theoretical model fit the empirical data sufficiently. Additional questions addressed whether the empirical data support the hypotheses stated above. Numerous studies point out that a preference for involvement in medical decisions is influenced by demographic characteristics like age, sex and education, [14-18], as much as by clinical characteristics like physical and mental health status or quality of life [7,17]. To control for their possible confounding effects, the influence of the following known measures on the central constructs was also modelled: demographic characteristics (age, sex, education), clinical characteristics (cardiovascular, musculoskeletal, or endocrinological disease), and quality of life (mental and physical). Additionally, the type of the administered medical treatment (diagnostics, therapy, or referral) was also considered.

## Methods

### Source of data

Cross-sectional data were collected from a study called “Gesundes Kinzigtal” (Engl. Healthy Kinzigtal), which evaluated an integrated healthcare system [19]. The “Kinzigtal” is a rural region in the Black Forest in Southern Germany. The main objective of the “Kinzigtal” project was to improve care through an intensive networking of different health care providers and institutions (integrated care). Another special focus of the project was to increase patient involvement in clinical decisions. The “Kinzigtal” project investigated one intervention and two control groups.

All insurants of the Health Insurance Fund AOK Baden-Württemberg and the Health Insurance Fund LKK Baden-Württemberg residing in the area of the Kinzigtal were suitable for participation in the study as members of the intervention or the first control group. The intervention group consisted of all insurants that were taking part in the “Kinzigtal” project at the reference date of 31.03.2007. The first control group consisted of insurants living in the same area but not taking part in the project. The second control group consisted of insurants living in an area that is comparable to the Kinzigtal with respect to healthcare infrastructure, population density, and local economy. To ensure that the control groups are comparable to the intervention group regarding central characteristics, insurants in these groups were selected by stratification of sex, age, kind of insurance, and healthcare costs they induced in the previous 12 months. All selected insurants were invited to participate in the study by a written invitation sent by their Health Insurance Fund (a more detailed description available in [19]).

The primary objective of the “Kinzigtal” project was to measure the effects of the integrated healthcare system on patient involvement and satisfaction with the physician.

We used data from the “Kinzigtal” project for the evaluation of the conceptual model. Our analysis is based on the pooled baseline data of all three groups mentioned above. The study was conducted in accordance with the principles of the Helsinki Declaration and with the approval of the ethics committee of the University Medical Center Freiburg (157/07).

### Data collection

Participants completed a postal questionnaire to allow for an assessment of relevant SDM-related constructs (see Table 2): preference for involvement in medical decisions, experienced involvement, decisional conflict, and satisfaction with the physician. All data were collected with reference to the last clinical encounter of the patient with a physician delivering outpatient care. Experienced involvement and decisional conflict were linked to a certain decision made during this encounter. On the contrary, preference for involvement and satisfaction with the physician were measured as generic constructs.

**Table 2:** Postal questionnaire

**Constructs**	**Measures**
Socio-demographic data	age; gender; native language; family status; partnership; education; occupation
Quality of Life	Health Survey SF-12 [20]
Clinical appointment	indication of appointment; time since appointment; subject of decision; decision made
Preference for involvement in medical decision making	Autonomy-Preference-Index (API; [21])
Experienced involvement	Shared Decision Making Questionnaire (SDM-Q-9; [22])
Decisional conflict	Decisional Conflict Scale (DCS; [23])
Patient satisfaction	Satisfaction with ambulatory care (ZAPA; [24])

The following instruments were used to assess the constructs:

The *Autonomy-Preference-Index* measures patients’ preferences regarding information and involvement in treatment decision-making [21,25]. It consists of 14 items, of which 8 items evaluate the need for information, and 6 items relate to the preference for involvement. In the present study, only the preference for involvement items were analysed. The internal consistency of both the original English and adapted German version is satisfactory with a Cronbach’s α of 0.82 and 0.86, respectively [21,25].

The 9-item-*Shared Decision-Making Questionnaire* (SDM-Q-9) assesses the extent of patient involvement in the decision-making process from the patient’s perspective. The internal consistency of the instrument is high, yielding a Cronbach’s α of 0.94 [22].

The *Decisional Conflict Scale* (DCS) measures uncertainty in making a health-related decision, factors contributing to the uncertainty, and effective decision-making [23,26]. Considering these many different facets, the construct of decisional conflict is quite heterogeneous. It includes 16 items. Both the internal consistency (Cronbach’s α = 0.78) and test-retest reliability (0.81) of the DCS are high.

To measure the patient’s satisfaction with the physician, we adopted an existing questionnaire (ZAP, [27]). We used two global items to measure the patient’s satisfaction with his or her outpatient care. We added one item to measure the patient’s global satisfaction with the quality and extent of information the patient received and another item to assess the patient’s global satisfaction with his or her involvement in clinical decisions. The resulting scale (ZAPA) had a satisfactory internal consistency with a Cronbach’s α of 0.90 [24].

Health-related *quality of life*, which was accounted for as a covariate, was captured with the SF-12 health survey [20]. The SF-12 is a psychometrically sound abbreviated version of the SF-36.

Demographic characteristics were assessed via closed multiple choice items. The patients reported their health complaints and reasons for consultation in an open response format (free text boxes).

### Data analysis

A path analysis using structural equation modelling [28,29] was employed to explore multiple associations. Data were analysed with AMOS 5 (SPSS Inc., Chicago, Illinois). The elements of the conceptual model were included in the model as defined a priori based on our theoretical assumptions. To control for their possible confounding effects, the influence of the following known measures on the central constructs was also modelled: demographic characteristics (age, sex, education), clinical characteristics (cardiovascular, musculoskeletal, or endocrinological disease), quality of life (mental and physical), and type of the clinical decision to be made (diagnostics, therapy, or referral). As these elements are not part of the conceptual model, their effects were freely estimated to fit the empirical data of these elements. A satisfactory model was developed in a development sub-sample and cross-validated in a confirmatory sample (split-half method). The split between the two samples was conducted by randomisation. The sub-sample in which the model was developed consisted of n = 983 patients; the confirmatory sub-sample comprised n = 930 patients.

Model development was performed in a stepwise process. First, a full path model was developed and included the following:

•all associations of central interest according to the model (Figure 1),

•all possible additional direct associations between the constructs (effect of involvement preference on decisional conflict and on patient satisfaction with the physician, as well as the effect of experienced involvement on patient satisfaction),

•all possible causal effects of confounders on SDM-related constructs, and

•all possible correlations between confounders.

Second, all correlations between confounders below 0.1 were eliminated, and the model was reassessed. Third, all clinically irrelevant causal associations (standardised regression coefficients below 0.1) were excluded, and the model was re-calculated. These steps were repeated until all of the correlation coefficients and standardised regression coefficients in the model exceeded 0.1.

The measurement model (the extent by which reliable constructs were measured) was assessed but not modified throughout the analysis because all of the included instruments proved to be psychometrically sound in previous studies and because the primary objective of the study was to examine associations between the SDM-related constructs and not to improve their measurement.

To describe the measurement model, local goodness-of-fit indexes were calculated. To describe the whole model, global goodness-of-fit measures were assessed [30-34].

An additional sensitivity analysis was conducted to test for possible model differences among the investigated groups. In this analysis, we fitted the statistical model to each of the three groups separately and investigated group differences with regard to the standardized regression weights. Thus, the consistency of our results (that were based on the pooled sample of the three groups) was tested additionally in the three original subgroups.

## Results

### Sample

For the data collection, 6,542 primary care patients were contacted in July 2007. Of the 2,450 patients (37.5%) who returned the questionnaire, a total of 1,913 complete data sets could be identified. The demographic and clinical characteristics, type of decision, and quality of life of the sample are presented in Table 3. Further information on descriptive results is given elsewhere [19]. These results showed comparable demographic and clinical characteristics for the intervention and the two control groups [19].

**Table 3:** Characteristics of the sample

	**Total sample**	**Development sub-sample**	**Confirmatory sub-sample**
*Sample size*	1,913	983	930
*Sex*			
Female	1,099 (57.5%)	551 (56.1%)	548 (58.9%)
Male	813 (42.5%)	432 (43.9%)	381 (41.0%)
*Age (yrs.)*			
Mean (SD) ^a^	62.1 (15.4)	62.5 (15.3)	61.8 (15.4)
*Family Status*			
Single	159 (8.5%)	80 (8.3%)	79 (8.7%)
Divorced	99 (5.3%)	48 (5.0%)	51 (5.6%)
Married	1,303 (69.9%)	672 (70.1%)	631 (69.7%)
Widowed	303 (16.3%)	159 (16.6%)	144 (15.9%)
*Education*			
Low	1,471 (78.4%)	749 (77.5%)	722 (79.3%)
Medium	300 (16.0%)	157 (16.2%)	143 (15.7%)
High	106 (5.6%)	61 (6.3%)	45 (4.9%)
*Employment status*			
Employed	563 (30.2%)	289 (30.0%)	274 (30.4%)
Retired	1,053 (56.5%)	556 (57.7%)	497 (55.2%)
Housewife	175 (9.4%)	85 (8.8%)	90 (10.0%)
Other^b^	72 (3.8%)	33 (3.3%)	39 (4,3%)
*Most frequent health complaints*			
Cardiovascular disease	397 (22.1%)	220 (24.0%)	177 (20.2%)
Muscoskeletal disease	584 (32.5%)	300 (30.5%)	284 (32.4%)
Endocrinological disease	198 (11.0%)	94 (10.2%)	104 (11.9%)
*Type of decision*			
Diagnostics	307 (17.1%)	159 (17.3%)	148 (16.9%)
Therapy	945 (52.6%)	498 (54.2%)	447 (51.0%)
Referral	445 (24.8%)	226 (24.6%)	219 (25.0%)
*Physical quality of life*^c^			
Low	715 (54.4%)	370 (55.1%)	345 (53.6%)
Normal	472 (35.9%)	236 (35.2%)	236 (36.6%)
High	128 (9.7%)	65 (9.7%)	63 (9.8%)
*Mental quality of life*^c^			
Low	616 (46.8%)	316 (47.1%)	300 (46.6%)
Normal	498 (37.9%)	249 (37.1%)	249 (38.7%)
High	201 (15.3%)	106 (15.8%)	95 (14.8%)

^a:^ standard deviation; ^b:^ self employed, students, unemployed, military service, not elsewhere classified; ^c:^ according to norm values of the SF-12.

### Model development and testing

Local goodness-of-fit indexes are displayed in Table 4. Factor loadings exceeded 0.4 in all scales. Both construct reliabilities (i.e., internal consistency) and the overall amount of variance in the indicators accounted for by the constructs reached recommended thresholds and indicated reliable measures.

**Table 4:** Local goodness-of-fit indexes

**Construct**	**Instrument**	**Factor loadings**	**Construct reliability**	**Variance extracted**
	*Thresholds for acceptable fit*	*≥0.4*	*≥0.6*	*≥0.5*
*Preference for involvement in medical decisions*	Autonomy Preference Index (API)	0.48 to 0.79	0.79	0.51
*Experienced involvement in medical decisions*	Shared Decision-Making Questionnaire (SDM-Q-9)	0.69 to 0.87	0.94	0.64
*Decisional conflict*	Decisional Conflict Scale (DCS)	0.67 to 0.87	0.96	0.62
*Satisfaction with physician*	ZAPA	0.78 to 0.89	0.92	0.71
*Mental quality of life*	SF-12 mental scale	0.66 to 0.74	0.84	0.50
*Physical quality of life*	SF-12 physical scale	0.71 to 0.81	0.89	0.60

recommendations are based on: [20,23,24,27,28].

Global goodness-of-fit indexes for each step of the model development are displayed in Table 5.

**Table 5:** Global goodness-of-fit indexes

	**Χ**^**2**^	**df**	**p**	**Χ**^**2**^**/d.f.**	**RMSEA**	**TLI**	**CFI**
*Recommendation for good fit*				*<2.0*	*< 0.05*	≥ *0.95*	≥ *0.95*
*Recommendation for acceptable fit*				*<5.0*	*<0.08*	≥ *0.90*	≥ *0.90*
**Model development**							
Step 1	5,594.09	1,346	<.001	4.156	0.057	0.860	0.878
Full path model							
Step 2	5,686.63	1,386	<.001	4.103	0.056	0.862	0.876
Correlations <0.1 removed							
Step 3	5,691.09	1,387	<.001	4.103	0.056	0.862	0.876
Correlations <0.1 removed							
Step 4	5,746.15	1,414	<.001	4.064	0.056	0.864	0.875
Causal associations <0.1 removed							
Step 5	5,749.91	1,415	<.001	4.064	0.056	0.864	0.875
Causal associations <0.1 removed							
Final model	5,271.38	1,066	<.001	4.945	0.063	0.863	0.876
Redundant variables removed							
**Confirmatory testing**							
Final model	5,064.57	1,066	<.001	4.751	0.064	0.860	0.873

*Df* degrees of freedom, *RMSEA* Root Mean Square Error of Approximation, *TLI* Tucker-Lewis Index, *NFI* Normed Fit Index; recommendations are based on: [20,23,24,27,28].

In total, the local and global fit indexes suggest an acceptable fit between the theoretical model and the data. In the cross-validation model, the Χ^2^-test proved to be statistically significant, indicating a difference between the theoretical model and the observed data. The normed Χ^2^ value was 4.751 and thus turned out to be acceptable. The Root Mean Square Error of Approximation yielded .064 and thus indicated a reasonable fit between the observed relations and the theoretical model. Both the Tucker-Lewis Index and the Comparative Fit Index were below the recommended value of ≥ .90 and therefore suggest that the model does not fit the data perfectly.

**Figure 2:**
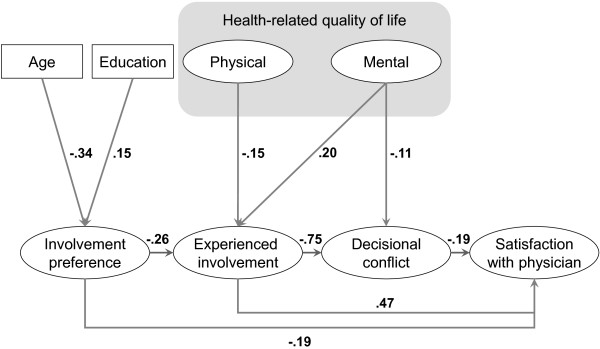
Path model in the developmental sample; displayed numbers are standardised regression coefficients; 0.1 = small effect, 0.3 = medium effect, 0.5 strong effect.

**Figure 3:**
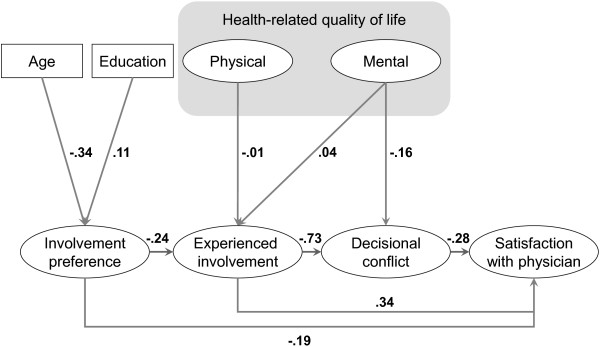
Path model in the confirmatory sample; displayed numbers are standardised regression coefficients; 0.1 = small effect, 0.3 = medium effect, 0.5 = strong effect.

The path model is displayed in Figures 2 and 3. The collected empirical data support most of the postulated associations between the SDM-related constructs in the model. In the cross-validation data set, increased *patient involvement* is strongly associated with decreased *decisional conflict* (standardised regression coefficient Β = −.73). Both *high involvement* and *low decisional conflict* predict higher *satisfaction with the physician* (the associations were Β = .34 and -.28, respectively).

The result that is most inconsistent with the model is the negative association of -.24 (p = <.001) between the patients’ *preference for involvement* and experienced *involvement*.

In considering possible confounders, a negative association of -.34 (p = <.001) was found between *age* and *preference for involvement*, suggesting that with increasing age, patients have a lower preference for involvement. *Education* and *preference for involvement* showed a weak positive association of .11 (p = .002), indicating that higher education is associated with a higher preference for involvement. Altogether, the interrelations between *quality of life* and the SDM-related constructs were relatively weak. The associations between *physical and mental health-related quality of life* and *experienced involvement* were -.01 (p = .876) and .04 (p = .545), respectively, and the association between *mental quality of life* and *decisional conflict* was -.16 (p = <.001). Neither of the covariates of *sex*, *clinical characteristics* (cardiovascular, musculoskeletal or endocrinological disease), or *type of decision to be made* (diagnostic, therapy or referral) showed an association > .1 with either of the SDM-related constructs and therefore were excluded from the model in the iterative process of model development.

The results of our sensitivity analyses are displayed in Additional files 1 and 2. Group differences with regard to the standardized regression weights were all below 0.20.

## Discussion

In the present study, a model of shared decision-making was tested via path analysis. The a priori postulated associations between different SDM-related constructs were examined in a sample of primary care patients. Furthermore, potential confounders (e.g., age, sex, education) of these SDM-related constructs were considered.

The examination of *global goodness-of-fit indexes* indicated an acceptable congruence between the model and the observed data. Although the index Χ^2^ turned out to be significant and thus suggested a difference between the model and the observed data, the sample sizes were so large that the significance of the Χ^2^-test is of little information [33,34]. Furthermore, the Tucker-Lewis Index and Comparative Fit Index were below the recommended value of .90 and thus showed a non-sufficient match between the data and model. However, normed Χ^2^ values and the Root Mean Square Error of Approximation indicated an acceptable fit. In summary, it can be concluded that the global goodness-of-fit indexes, though not optimal, support the plausibility of the proposed model. It is possible that a data-driven modification of the measurement model and a post hoc adaptation of the instruments (e.g., through allowing for correlated error terms between items within the scales) would have led to a better global fit. However, refining the measurements of the examined constructs was not the focus of this investigation. As the concept of SDM is still relatively new, both the refinement of existing instruments and the development of new instruments measuring SDM-related constructs are necessary [12,35-37].

Altogether, our theoretical assumptions could be largely corroborated by the collected empirical data.

Consistent with our hypothesis, higher patient involvement clearly lowered decisional conflict. The effect was quite high, and the finding is consistent with the literature [38,39]. Therefore, the postulated effect between the process and an intermediate endpoint of SDM could be confirmed. A result that was inconsistent with our expectations was the negative association between the patients’ *preference for involvement* and their current *involvement*. In the model, it was assumed that patients who wish to be involved in decision-making would actually be more strongly involved. In the empirical data, the opposite was found. A possible explanation could be that those patients who had a strong involvement preference had high expectations concerning their involvement and thus experienced the actual involvement as unsatisfactory. This explanation could also account for the negative association between *preference for involvement* and *satisfaction* with the physician. It is possible that those patients who had high expectations concerning their involvement evaluated their consultation and their physician more critically. Another explanation could be that *preference for involvement* is highly subjective depending on the context and circumstances [40]. Thus, involvement preference in the specific medical encounter might be very different from the generic *preference for involvement in decision-making*. These results highlight the importance of collecting data on patient preferences for involvement in addition to the assessment of the experienced involvement to be able to assess the concordance between these measures.

*Satisfaction with the physician* was clearly affected by *patient involvement* and *decisional conflict*. An increased patient involvement affected satisfaction with the physician directly and indirectly through decreased decisional conflict. Thus, decisional conflict may be considered as a mediator between involvement and satisfaction with the physician. The direct and indirect effects of *involvement* on *satisfaction with the physician* summed up to a large effect (.54) in the path model. This result is highly consistent with the results of Quaschning et al. [41] who used a very similar approach. In line with our results, they could explain a high proportion of variance in *patient satisfaction* by *patient involvement. Satisfaction with decision* proved to mediate the effect of patient involvement on *patient satisfaction*. As *satisfaction with decision* is a construct that can - just like *decisional conflict* - be categorized as decision outcome [10] this result is very close to our findings.

The findings were relatively independent of covariates. The most affected of all the constructs was the *preference for involvement*. Both higher age and lower educational status are known to decrease preference for involvement [18]. In accordance with these findings, a medium influence of age and a moderate influence of education on involvement preference were found in this study. Both physical and mental health-related quality of life covariates were moderately associated with the SDM-related constructs *experienced involvement* and *decisional conflict* in the development sample. In the confirmatory sample, only the interrelation between mental quality of life and decisional conflict proved to be relevant. Thus, a low mental quality of life is possibly associated with increased decisional conflict. For the covariates that dropped out in the iterative process of model development (sex, clinical characteristics, and type of decision), it can be assumed that they may not have a substantial effect on the constructs in the examined population.

When interpreting the present results, some limitations should be taken into account. First, on average, the examined sample was of older age and of low-educational level and rural origin. Second, due to the cross-sectional design of the study, the examined associations are correlative and not necessarily causal. The temporal relationships in the model had to be assumed and cannot be confirmed using cross-sectional data. However, these assumptions may be supported by the instructions applied in the study. For example, for data collection on SDM, participants were instructed to rate physician and conjoint behaviour during the decision-making process, while for decisional conflict, they were asked for their experiences after decision-making. Third, the results of our study may also be influenced by the limitations of memory, as patients had to remember their last clinical encounter. Furthermore, all sources of data are derived from the same questionnaire, what might lead to common methods bias. Although the intervention and the two control groups did not differ substantially either regarding demographic and clinical characteristics in baseline comparisons or regarding associations of the investigated constructs in sensitivity analyses, the use of the pooled baseline data might still have introduced some unobserved bias.

As data on the treating physicians were not available, we were unable to account for the hierarchical structure of our data due to patients clustered by physician. This is a serious limitation and may have led to an underestimation of the standard error of our parameters. Additionally, our model only considers one of many possible intermediate and long-term endpoints. With respect to our instruments, it should be taken into account that decisional conflict considers many different facets and thus is quite heterogeneous. Therefore, results on this scale should be interpreted with caution.

Future research on the associations among different SDM-related constructs should incorporate longitudinal data from intervention studies. By using this design, causal effects of changes in patient involvement on decisional conflict and satisfaction with the physician can be clarified. The role of patient preferences for involvement in the process remains poorly understood. Further investigation on this topic is needed, as it is a central construct in recent debates on indications for applying shared decision-making. More empirical evidence on the associations among the SDM-related constructs could lead to a better understanding of the decision-making process. Other constructs of SDM could be incorporated in future investigations. Based on the model, nomological networks for the validation of psychometric instruments could be developed and tested. A better theoretical foundation of SDM could be helpful for increasing the implementation of this promising concept into health care. The model could also help to choose adequate outcome parameter for studies of shared decision making (e.g. a study focusing on patient preferences would need other instruments than a study with focus on the process).

## Conclusion

Altogether, our model could be largely corroborated by the collected empirical data. *Satisfaction with the physician* was clearly affected by *patient involvement* and *decisional conflict*. An increased patient involvement affected satisfaction with the physician directly and indirectly through a decreased decisional conflict. Thus, decisional conflict may be considered as a mediator between involvement and satisfaction with the physician. Direct and indirect effects of *involvement* on *satisfaction with the physician* summed up to a large effect (.54) in the path model.

## Competing interests

The authors declare that they have no competing interests.

## Authors’ contributions

LPH, LK and MH developed the conception and design of the study. LPH, LK and MH collected data. LK and LPH undertook the statistical analysis. All authors contributed to the interpretation of results. All authors have approved the final manuscript.

## Pre-publication history

The pre-publication history for this paper can be accessed here:

http://www.biomedcentral.com/1472-6963/13/231/prepub

## Supplementary Material

Additional file 1 **Global goodness-of-fit indexes of subgoups **[24,25]**.** Click here for file

Additional file 2 Standardized regression weights of subgroups. Click here for file
