# Supplementary material for: Patient preference for involvement, experienced involvement, decisional conflict, and satisfaction with physician: a structural equation model test
Source: BMC Health Serv Res. 2013 Jun 25;13:231. doi: 10.1186/1472-6963-13-231 (PMC3701592; doi:10.1186/1472-6963-13-231)
Supplement: Additional file 1 — Global goodness-of-fit indexes of subgoups [24,25]. [file 1472-6963-13-231-S1.doc]

**Additional File 1. Global goodness-of-fit indexes of subgoups**

|  | **Χ^2^** | **df** | **p** | **Χ^2^/d.f.** | **RMSEA** | **TLI** | **CFI** |
| --- | --- | --- | --- | --- | --- | --- | --- |
| *Recommendation for good fit* |  |  |  | *<2.0* | *< 0.05* | *≥ 0.95* | *≥ 0.95* |
| *Recommendation for acceptable fit* |  |  |  | *<5.0* | *<0.08* | *≥ 0.90* | *≥ 0.90* |
| **Development** | 5,271.38 | 1,066 | <.001 | 4.945 | 0.063 | 0.863 | 0.876 |
| **Test** | 5,064.57 | 1,066 | <.001 | 4.751 | 0.064 | 0.860 | 0.873 |
| **Intervention Group (N = 395)** | 2,779.50 | 1,066 | <.001 | 2.607 | 0.064 | 0.859 | 0.872 |
| **Control Group 1 (N = 627)** | 4,111.29 | 1,066 | <.001 | 3.857 | 0.068 | 0.851 | 0.865 |
| **Control Group 2 (N = 757)** | 4,455.21 | 1,066 | <.001 | 4.179 | 0.065 | 0.853 | 0.867 |

df = degrees of freedom; RMSEA = Root Mean Square Error of Approximation; TLI = Tucker-Lewis Index; NFI = Normed Fit Index; recommendations are based on:[24-28]
