# Supplementary material for: Patient preference for involvement, experienced involvement, decisional conflict, and satisfaction with physician: a structural equation model test
Source: BMC Health Serv Res. 2013 Jun 25;13:231. doi: 10.1186/1472-6963-13-231 (PMC3701592; doi:10.1186/1472-6963-13-231)
Supplement: Additional file 2 — Standardized regression weights of subgroups. [file 1472-6963-13-231-S2.doc]

**Additional File 2. Standardized regression weights of subgroups**

| **Variables** | **Estimates** | | | | | |
| --- | --- | --- | --- | --- | --- | --- |
|  | Development | Test | IG | CG1 | CG2 | Δ max. |
| IP – age | -.34 | -.34 | -.32 | -.38 | -.33 | .06 |
| IP – education | .15 | .11 | .16 | .15 | .08 | .08 |
| EI – IP | -.26 | -.24 | -.26 | -.29 | -.20 | .09 |
| EI– physical health | -.15 | -.01 | -.10 | -.10 | -.07 | .14 |
| EI–mental health | .20 | .04 | .18 | .17 | .07 | .16 |
| DC – EI | -.75 | -.73 | -.74 | -.71 | -.76 | .05 |
| DC – mental health | -.11 | -.16 | -.14 | -.17 | -.14 | .06 |
| SP – DC | -.19 | -.28 | -.32 | -.24 | -.19 | .13 |
| SP – EI | .47 | .34 | .38 | .39 | .43 | .13 |
| SP – IP | -.19 | -.19 | -.13 | -.19 | -.20 | .07 |

IG = intervention group; CG1 = control group 1; CG2 = control group 2; Δ max. = maximum difference; IP = involvement preference; EI = experienced involvement; DC = decisional conflict; SP = satisfaction with physician
